# Rapid antigen tests for the detection of SARS-CoV-2: A narrative review

**DOI:** 10.1016/j.aprim.2021.102127

**Published:** 2021-05-28

**Authors:** Antonio L. Aguilar-Shea, Mar Vera-García, Robert Güerri-Fernández

**Affiliations:** a Centro de Salud Puerta de Madrid, Atención Primaria de Madrid, Spain; b Hospital Clinical Multiprofesional COVID19 Unit, Avda del Ejército 61, 28802 Alcalá de Henares (Madrid), Spain; c Centro Sanitario Sandoval, Calle de Sandoval, 7, 28010 Madrid, Spain; d HIV/STD Unit, Hospital Clínico Universitario San Carlos, IdISSC, Calle del Profesor Martín Lagos, s/n, 28040 Madrid, Spain; e Hospital del Mar - Parc de Salut MAR, Paseo Marítimo de la Barceloneta, 25-29, 08003 Barcelona, Spain

**Keywords:** SARS-CoV-2, COVID-19, Antigen test, Rapid, Diagnosis, Point-of-care, SARS-CoV-2, COVID-19, Test de antígenos, Rápido, Diagnóstico, Centró de atención médica

## Abstract

The rapid identificationand isolation of COVID-19 patients has become the cornerstone for the control of the recent outbreak. Real-time quantitative polymerase chain reaction is routinely used to confirm COVID-19 diagnosis and is considered the gold standard due to high sensitivity and specificity. Nevertheless, it usually takes several days and a relatively higher cost. Antigen tests based have emerged to cope with such disadvantages, by offering rapid results, an easy-to-use procedure, and low costs. The objective of the narrative review was to provide up-to-date data about CE-marked rapid antigen tests (RATs) for COVID-19. Given their large number, the study only focused on representative and widely used in Spain (Standard Q, Nadal, Panbio, CerTest, and Wondfo). RATs have become a very useful and validated tool for controlling the spread of COVID-19 allowing the rapid identification of active infection and isolation of positive patients. The present revision of the literature has demonstrated that sensitivity and specificity of all available RATs in Spain are high and accomplish European regulations and WHO recommendations.

## Abstract (es)

La rápida identificación y aislamiento de los pacientes por COVID-19 se ha convertido en un reto para el control de la pandemia. La reacción en cadena de la polimerasa cuantitativa a tiempo real se utiliza de manera rutinaria para confirmar el diagnóstico de COVID-19 y se considera el estándar de referencia por su alta sensibilidad y especificidad. Sin embargo, normalmente lleva varios días y los costes son relativamente altos. Los test de antígeno son una herramienta rápida, fácil de realizar y de bajo coste. El objetivo de la revisión narrativa es proporcionar información actualizada sobre los test rápidos de antígenos (TRA) para COVID-19 con marcado europeo. Dado su gran número, el estudio solo se enfoca en aquellos representativos y ampliamente empleados en España (Standard Q, Nadal, Panbio, CerTest y Wondfo). Los TRA se han convertido en una herramienta validada muy útil para controlar la diseminación de la COVID-19, al permitir la identificación rápida de la infección activa y el aislamiento de pacientes positivos. La presente revisión de la literatura ha demostrado que la sensibilidad y especificidad de los TRA disponibles en España son altas y cumplen con la regulación europea y las recomendaciones de la OMS.

## Introduction

Coronaviruses are an important family of pathogenic agents that affect the human respiratory system, and were responsible for worldwide outbreaks such as the severe acute respiratory syndrome (SARS)-CoV in 2003, or the Middle East respiratory syndrome (MERS)-CoV in 2012.^1^, ^2^ By December 2019, a cluster of atypical pneumonia cases were reported in Wuhan (China). The etiological agent was identified as a severe acute respiratory syndrome coronavirus 2 (SARS-CoV-2), and its related disease was named as coronavirus disease 2019 (COVID-19).^3^ The disease shows diverse clinical manifestations, i.e. from no or mild symptoms to severe pneumonia with multi-organ failure, including acute respiratory distress syndrome, sepsis, and septic shock.^4^ On 30^th^ January 2020 the World Health Organization (WHO) declared COVID-19 outbreak to be a public health emergency of international concern^5^; the sixth after H1N1 (2009), Polio (2014), Ebola in West Africa (2014), Zika (2016) and Ebola in the Democratic Republic of Congo (2019).^6^ The first case of COVID-19 in Spain was confirmed on 31^st^ January 2020.^7^ Since then, a total of 3,096,343 individuals have been tested positive, and 65,979 ones have passed away.^8^ The rapid identification and isolation of positive patients has become the cornerstone for the control of the COVID-19 outbreak.^9^ Real-time quantitative polymerase chain reaction (RT-qPCR), with nasopharyngeal swabs, throat swabs, or saliva samples, is routinely used to confirm the COVID-19 diagnosis.^10^ It is considered the gold standard due to its high sensitivity and specificity.^11^ Nevertheless, RT-qPCR testing usually takes several days and relatively higher costs to be completed. It requires the transportation of samples from the place of collection to the specialized laboratory, 4–6-h of methodology, limited laboratory capacity (trained staff, special equipment, reagents, disposables, etc.), and high sample volumes.^11^ Antigen tests based on lateral flow immunoassays have emerged to cope with such a disadvantage scenario, by offering rapid results (10–30 min), through an easy-to-use procedure, and low costs. By December 2020, the European Commission purchased over 20 million rapid antigen tests (RATs) for European members, and stated recommendations for their use and validation.^12^ The objective of the present narrative review was to provide up-to-date data about RATs for COVID-19 with European conformity (*CE* marked). Given their large number,^13^ the study only focused on representative and widely used in Spain, i.e. Standard Q (SD Biosensor Inc., Republic of Korea; F. Hoffmann-La Roche Ltd, Switzerland), Nadal (Nal von minden GmbH, Germany), Panbio (Abbott Laboratories GmbH, Germany), CerTest (Certest Biotec, S.L., Spain), and Wondfo (Guangzhou Wondfo Biotech Co. Ltd, China).

## Methods

A systematic review of available studies involving RATs was carried out in PubMed database. Keywords included: “antigen test”, “COVID-19”, “SARS-CoV-2”, “Standard Q”, “Roche”, “Nadal”, “Panbio”, “Abbott”, “CerTest”, and “Wondfo”. The date of search was February 18^th^ 2021. Only studies providing information about sensitivity and specificity of RATs were analyzed. With the aim of including more studies (especially preprint papers, studies that have not been published on PubMed, or information from manufacturers), manual searches were carried out in Google by using the same keywords.

## Standard Q COVID-19 antigen test

The Standard Q test was the second commercialized RAT in Spain. It detects the presence of several SARS-CoV-2 nucleocapsid proteins in nasopharyngeal swabs within 15–30 min.^14^ Initial validation studies of the Standard Q test for the diagnosis of SARS-CoV-2 infection were conducted by Foundation for Innovative New Diagnostics (FIND), a global non-profit organization involved in the development and delivery of diagnostics, in 1659 subjects from 2 cohorts of Germany and one from Brazil.^14^, ^15^ Samples included nasopharyngeal and oropharyngeal specimens. Of subjects, 9.2% were RT-qPCR positive for SARS-CoV-2. Overall sensitivity and specificity of the RAT was 85.0% (95% CI: 78.3–90.2) and 98.9% (95% CI: 98.2–99.4), respectively. Diverse studies have subsequently evaluated the use of Standard Q test in cohort of subjects (Table 1).^16^, ^17^, ^18^, ^19^, ^20^, ^21^, ^22^, ^23^, ^24^, ^25^ Chaimayo et al.,^17^ studied 454 respiratory samples from symptomatic patients and close contacts and reported 13.2% of prevalence of SARS-CoV-2 infection by RT-qPCR. The median onset of symptoms in patients was 3 days. Of samples, 13.0% tested positive with the Standard Q test. Overall sensitivity and specificity were 98.3% (95% CI: 91.1–100.0), and 98.7% (95% CI: 97.1–99.6). Five false positives, out of 394 RT-qPCR negative tests (1.3%), were reported. Similarly, Cerutti et al.,^20^ evaluated the Standard Q test in 330 subjects (185 symptomatic patients) referred at the emergency rooms of two Italian Centers. The detection rate with RT-qPCR was 33.0%, and 23.3% with the RAT. No false positives were identified. Thus, overall sensitivity, specificity, PPV, and NPV were 70.6%, 100.0%, 100.0%, and 87.4%, respectively.

**Table 1:** Summary of studies involving the Standard Q test.

Reference	Publication type	Population	RT-qPCR positive	Ag test positive	Overall sensitivity (95% CI)	Ag test false positive	Overall specificity (95% CI)	NPV (95% CI)	PPV (95% CI)
FIND^14^, ^15^	Non-published, public results	1659	153/1659 (9.2%)	NA	85.0% (78.3–90.2)	NA	98.9% (98.2–99.4)	NA	NA
Nalumansi et al.,^16^	Research article	262 (172 ASNCP)	90/262 (34.4%)	76/262 (29.0)	70% (60–79)	8% (95% CI: 4–13)	92% (87–96)	NA	NA
Chaimayo et al.,^17^	Research article	454 (SP and ASCP)	60/454 (13.2%)	59/454 (13.0%)	98.3% (91.1–100.0)	5/394 (1.3%)	98.7% (97.1–99.6)	NA	NA
Mak et al.,^18^	Research article	35 samples	NA	NA	60%, 71.4%, 65.7%, and 71.4%^a^	NA	NA	NA	NA
Berger et al.,^19^	Preprint (not peer-reviewed)	529	191/529 (36.1%)	170/529 (32.1%)	89.0% (83.7–93.1)	1/338 (0.3%)	99.7% (98.4–100.0)	94.1% (91.2–96.3)	99.4% (96.8–100.0)
Cerutti et al.,^20^	Short communication	330 (185 SP)	109/330 (33.0%)	77/330 (23.3%)	70.6%	0 (0.0%)	100.0%	87.4%	100.0%
Krüger et al.,^21^	Preprint (not peer-reviewed)	1263 (1901 SP)	47/1263 (3.7%)	45/1263 (3.6%)	76% (62.8–86.4)	9/1216 (0.7%)	99.3% (98.6–99.6)	NA	NA
Iglοi et al.,^22^	Preprint (not peer-reviewed)	970 (SP or ASCP)	186/970 (19.2%)	NA	84.9% (79.1–89.4)	NA	99.5% (93.8–99.0)	96.5% (95.0–97.6)	97.5% (94.0–99.5)
Krüttgen et al.,^23^	Short communication	150	75/150 (50.0%)	56/150 (37.3%)	70.7%	3/75 (4.0%)	96%	NA	NA
Schwob et al.,^24^	Preprint (not peer-reviewed)	333 SP	NA	NA	92.9% (86.4–96.9)	NA	100% (99.3–100.0)	NA	NA
Salvagno et al.,^25^	Research article	321	149/321 (46.4%)	101/321 (34.0%)	72.5% (64.6–79.5)	NA	99.4% (96.8–100.0)	NA	NA

RT-qPCR, real-time quantitative polymerase chain reaction; Ag, antigen; 95CI, 95% confidence interval; NPV, negative predictive value; PPV, positive predictive value; FIND, The Foundation for Innovative New Diagnostics; SP, symptomatic patients; ASCP, asymptomatic subjects in contact with patients; NA, not available; ASNCP, asymptomatic subjects not in contact with patients.

a Sensitivities calculated from nasopharyngeal aspirate and throat swabs; nasopharyngeal and throat swabs; nasopharyngeal swabs; and throat saliva, respectively.

## Nadal COVID-19 antigen test

Nadal antigen test is another RAT widely used in Spain.^26^ The test directly checks for the presence of specific SARS-CoV-2 nucleocapsid proteins in 15 min. According to the manufacturer's information, the diagnostic sensitivity is 80.2% (95% CI: 73.9–85.3%) for a Ct value 20–37 or 97.6% (95% CI: 93.1–99.2%; Table 2) if Ct value 20–30, and the diagnostic specificity is more than 99.9% (95% CI: 97.7–100.0).^26^ These data derive from a study with 348 samples, 46.3% being RT-qPCR positive, and 43.1% Nadal test-positive. No false positives with Nadal test were reported. Few studies have been published to evaluate the Nadal antigen test for the diagnosis of SARS-CoV-2 infection. All of them have compared the analytical sensitivity, and clinical sensitivity and specificity in diverse RATs (Table 3).^18^, ^27^, ^28^ For instance, Kohmer et al.,^28^ evaluated 100 nasopharyngeal swab samples, from subjects living in a shared facility, and reported 74.0% of SARS-CoV-2 infection by RT-qPCR. Of the 74 RT-qPCR positive samples, authors re-tested them with four RATs, and found positivity with Nadal in 18 (24.3%). Sensitivity and specificity with Nadal were 24.3% (95% CI: 15.1–35.7) and 100% (95% CI: 86.8–100.0), respectively.

**Table 2:** Summary of studies involving the Nadal test.

Reference	Publication type	Population	RT-qPCR positive	Ag test positive	Overall sensitivity (95% CI)	Ag test false positive	Overall specificity (95% CI)	NPV (95% CI)	PPV (95% CI)
Nal von minden^26^	Non-published, public results	348 samples	161/348 (46.3%)	150/348 (43.1%)	80.2% (73.9–85.3%) or 97.6% (93.1–99.2%)	0 (0.0%)	>99.9% (97.7–100.0)	NA	NA
Mak et al.,^18^	Research article	35 samples	NA	NA	100%, 100.0%, 100%, and 77.8%^a^	NA	NA	NA	NA
Strömer et al.,^27^	Research article	134 samples	124/134 (92.5%)	NA	79/108 (73.1%)^b^	NA	NA	NA	NA
Kohmer et al.,^28^	Research article	100 samples	74/100 (74.0%)	18/74 (24.3%)	24.3% (15.1–35.7)	NA	100% (86.8–100.0)	NA	NA

RT-qPCR, real-time quantitative polymerase chain reaction; Ag, antigen; 95CI, 95% confidence interval; NPV, negative predictive value; PPV, positive predictive value.

a Sensitivities calculated from nasopharyngeal aspirate and throat swabs; nasopharyngeal and throat swabs; nasopharyngeal swabs; and throat saliva, respectively.

b Sensitivity calculated for cycle threshold ≤30.

**Table 3:** Summary of studies involving the Panbio test in nasopharyngeal samples.

Reference	Publication type	Population	RT-qPCR positive	Ag test positive	Overall sensitivity (95% CI)	Ag test false positive	Overall specificity (95% CI)	NPV (95% CI)	PPV (95% CI)
Linares et al.,^31^	Short communication	184 SP and 67 ASCP (255 swabs)	60 (23.5%)	44 (17.2%)	73.3% (62.2–83.8)	0 (0.0)	NA	NA	NA
Albert et al.,^32^	Research note	412 SP	54/412 (13.1%)	43/412 (10.4%)	79.6% (67.0–88.8)	0 (0.0)	100.0% (98.7–100.0)	99% (97.4–99.6%) and 97.9% (95.9–98.9)^a^	NA
Alemany et al.,^33^	Letter to editor	446 SP, 473 ASCP, and 487 ASNCP	951 (67.6%)	872/951 (91.7%)	91.7% (89.8–93.4)	NA	98.9% (97.5–99.6)	99.6% (99.5–99.7)^b^	81.5% (65.0–93.2)^b^
Winkel et al.,^34^	Preprint (not peer-reviewed)	824 (2425 swabs)	52/824 (6.3%)	42/824 (5.1%)	From 61.8 (49.2–73.3) to 69.1% (56.7–79.8)	0 (0.0)	From 99.5% (99.2–99.8) to 100.0% (99.8–100.0%)	NA	NA
Bulilete et al.,^35^	Commentary	1369 (503 SP, 750 ASCP, 116 unknown)	140/1369 (10.2%)	102/1369 (7.5%)	71.4% (63.1–78.1)	2/1222 (0.1%)	99.8% (99.4–99.9)	96.8% (95.7–97.7)	98.0% (93.0–99.7)
Torres et al.,^36^	Research note	634 ASCP	79 (12.4%)	38/79 (48.1%)	48.1% (37.4–58.9)	0 (0.0%)	100.0 (99.3–100.0)	93.1% (90.8–94.9)^c^	100.0% (90.8–100.0)^c^
Fenollar et al.,^37^	Letter to editor	182 SP	182 (100%)	144/182 (79.1%)	75.5% (69.5–81.5)	0 (0.0%)	94.9% (91.2–98.6)	95.6%	72.2%
		159 ASCP	22/159 (13.8%)	10/22 (45.4%)		7/137 (5.1%)			
Villaverde et al.,^38^	Brief reports	1620 SP (pediatrics)	77/1620 (4.8%)	38/1620 (2.3%)	45.4% (34.1–57.2)	3/1543 (0.2%)	99.8% (99.4–99.9)	97.3% (96.8–97.8)^d^	92.5% (78.6–97.4)^d^
Gremmels et al.,^39^	Research paper	1367 SP in the Netherlands	139 (10.2%)	101/1367 (7.4%)	72.6% (64.5–79.9)	0 (0.0)	100.0% (99.7–100.0)	NA	NA
		208 SP in Aruba	63 (30.3%)	51/208 (24.5%)	81.0% (69.0–89.8)				
Domínguez Fernández et al.,^40^	Scientific letter	27 SP 3 ASCP	20/30 (66.7%)	19/30 (63.3%)	95%	0 (0.0%)	100%	90.9%	100.0%
Masiá et al.,^41^	Research paper	913 (296 ASCP)	196/913 (21.5%)	120/913 (13.1%)	94% (85–98) and 80% (67–85)^e^	0 (0.0%)	∼100%	NA	NA

RT-qPCR, real-time quantitative polymerase chain reaction; Ag, antigen; 95CI, 95% confidence interval; NPV, negative predictive value; PPV, positive predictive value; SP, symptomatic patients; ASCP, asymptomatic subjects in contact with patients; NA, not available; ASNCP, asymptomatic subjects not in contact with patients.

NPV and PPV were calculated for an estimated prevalence of:

a 5% and 10%, respectively;

b 5%;

c 12.4%;

d 4.8%.

e Values calculated for cycle threshold ≤25 and <30, respectively.

## Panbio COVID-19 antigen rapid test

Panbio was the first commercialized RAT for COVID-19 in Spain.^29^ The test detects the presence of a SARS-CoV-2 nucleocapsid protein in nasal or nasopharyngeal swab specimens within 15 min.^30^ According to the manufacturer's information, the sensitivity and specificity is: nasal swab versus nasal RT-qPCR (sensitivity: 98.1% or 99.0% for samples with cycle threshold, Ct, values ≤33; and specificity: 99.8%), nasal swab versus nasopharyngeal RT-qPCR (sensitivity: 91.1%; specificity: 99.7%), and nasopharyngeal swab versus nasopharyngeal RT-qPCR (sensitivity: 91.4% or 94.1% for samples with Ct values ≤33; specificity: 99.8%). Panbio has been validated in diverse studies (Table 3).^31^, ^32^, ^33^, ^34^, ^35^, ^36^, ^37^, ^38^, ^39^, ^40^, ^41^ Fenollar et al.^37^ evaluated Panbio in nasopharyngeal specimens from 182 symptomatic patients and 159 asymptomatic subjects who were in contact with patients (“close contacts”). Panbio detected 144 out of the 182 RT-qPCR confirmed symptomatic patients (79.1%) and 10 out of 22 RT-qPCR confirmed close contacts (showing Ct ≥ 25). False positives with the RAT were reported in 7 of the 137 RT-qPCR negative asymptomatic subjects (5.1%). Thus, overall sensitivity and specificity were 75.5% (95% confidence interval, 95% CI: 69.5–81.5) and 94.9% (95% CI: 91.2–98.6), respectively. Similarly, Linares et al.,^31^ in a study with 184 symptomatic patients and 67 asymptomatic close contacts (involving a total of 255 swabs), revealed an overall sensitivity with Panbio of 73.3% (95% CI: 62.2–83.8). No false positives were reported. Authors also demonstrated that patients with onset of symptoms <7 days showed a significantly higher viral load, and a greater sensitivity of the antigen test (86.5%, 95% CI: 75.5–97.5) than those with ≥7 days (53.8%, 95% CI: 26.7–80.9). Bulilete et al.,^35^ in another study with 1369 subjects attending primary healthcare (503 symptomatic patients, 750 asymptomatic close contacts, and 116 unknown), showed a SARS-CoV-2 prevalence of 10.2% (140 RT-qPCR confirmed patients). Onset of symptoms was predominantly within 5 days (70.6% of patients). Overall sensitivity, specificity, positive predictive value (PPV), and negative predictive value (NPV) were 71.4% (95% CI: 63.1–78.7), 99.8% (95% CI: 99.4–99.9), 98.0% (95% CI: 93.0–99.7), and 96.8% (95% CI: 95.7–97.7), respectively. Sensitivity was greater in symptomatic patients, with symptom onset within 5 days, and high viral load. Finally, Villaverde et al.,^38^ in a study with 1620 symptomatic pediatric patients, revealed an overall sensitivity of 45.4% (95% CI: 34.1–57.2), and a specificity of 99.8% (95% CI: 99.4–99.9). False positives were reported in 0.2% of cases (3 out of 1543 RT-qPCR negative tests).

## CerTest SARS-CoV-2 card test

CerTest test represents the first commercialized RAT that is manufactured by a Spanish Biotech company.^42^ It is also based on the qualitative detection of SARS-CoV-2 membrane proteins from nasopharyngeal swab samples. The only available information related to its clinical sensitivity and specificity derives from the leaflet of the manufacturer.^43^ It includes a comparison between CerTest SARS-CoV-2 card test and RT-qPCR in 262 nasopharyngeal samples from subjects with suspicion of infection (Table 4). The prevalence of infection by RT-qPCR was 10.7% (28 out of 262), and 10.3% (27 out of 262) by the RAT. One false positive, out of 234 RT-qPCR negative tests (0.4%), was reported. Therefore, the sensitivity was 92.9% (95% CI: 76.5–99.1), and the specificity was 99.6% (95% CI: 97.6–100.0). The NPV and PPV were 96.3% (95% CI: 81.0–99.9) and 99.1% (95% CI: 97.0–99.9).

**Table 4:** Summary of studies involving CerTest or Wondfo antigen tests.

Reference	Publication type	Population	RT-qPCR positive	Ag test positive	Overall sensitivity (95% CI)	Ag test false positive	Overall specificity (95% CI)	NPV (95% CI)	PPV (95% CI)
*CerTest*									
Certest Biotec^43^	Non-published, public results	262	28/262 (10.7%)	27/262 (10.3%)	92.9% (76.5–99.1)	1/234 (0.4%)	99.6% (97.6–100.0)	96.3% (81.0–99.9)	99.1% (97.0–99.9)

*Wondfo*									
Guangzhou Wondfo Biotech^45^	Non-published, public results	859	497/859 (57.9%)	479/859 (55.8%)	96.2% (96.4–98.5)	1/362 (0.3%)	99.7% (98.5–100.0)	NA	NA
FIND^46^	Non-published, public results	328	56/328 (17%)	NA	85.7% (74.3–92.6)	NA	100% (98.6–100.0)	NA	NA

RT-qPCR, real-time quantitative polymerase chain reaction; Ag, antigen; 95CI, 95% confidence interval; NPV, negative predictive value; PPV, positive predictive value; FIND, The Foundation for Innovative New Diagnostics.

## Wondfo 2019-nCoV antigen test

Wondfo test is another commercialized and widely used RAT in Spain. It qualitatively detects a SARS-CoV-2 nucleocapsid protein in nasopharyngeal/oropharyngeal samples within 15–20 min.^44^ Data on sensitivity and specificity also derive from the manufacturer and a couple of reports from FIND foundation (Table 4).^45^ A study with 859 oropharyngeal swab samples showed 497 RT-qPCR positive ones. Most of patients (92.9%) showed onset of symptoms within 0–5 days. Wondfo test showed a prevalence of 55.8%. One false positive was reported (0.3% of RT-qPCR negative cases). Sensitivity and specificity were 96.2% (95% CI: 96.4–98.5) and 99.7% (95% CI: 98.5–100.0), respectively. One of the FIND reports describes a study in the University Hospital of Geneva with 328 nasopharyngeal swab specimens.^46^ The prevalence of SARS-CoV-2 infection was 17% by RT-qPCR. The median days from symptom onset was 2 (range: 1–4). Sensitivity of Wondfo test was 85.7% (95% CI: 74.3–92.6%), and specificity was 100% (95% CI: 98.6–100.0).

## Strengths and limitations on rapid antigen tests

Overall clinical sensitivity of RATs has demonstrated to be high; however there is some variability among studies. By contrast, overall specificity is consistently elevated in all of them. RATs are especially adequate in patients with high viral loads (Ct values ≤25 or >106 genomic virus copies/mL) which frequently present symptoms within the first 5–7 days of the disease.^47^ However, symptomatic patients with onset of symptoms beyond 7 days are more likely to show lower viral loads, and thus the likelihood of false negative results with RATs is greater. According to a recent systematic review and meta-analysis with data from 52 studies that evaluated the accuracy of 20 different RATs, sensitivity and specificity are 73.8% (95% CI: 68.6–78.5) and 99.7% (95% CI: 99.3–99.9), respectively.^48^ The WHO established the minimum requirements of sensitivity (≥80%) and specificity (≥97%) for RATs to be used for the diagnosis of SARS-CoV-2 infection when RT-qPCR is unavailable or where prolonged turnaround times preclude clinical utility.^47^ Available information on sensitivity and specificity from RATs are relatively scarce and derive from studies with differential designs, participating subjects, and test brands being evaluated. Lateral flow assays are point-of-care formats, and involve simple and easy-to-produce devices.^49^ RATs can also provide a result in minutes, in contrast to the few days required with RT-qPCR.

## Conclusions

Antigen tests have become a very useful and validated tool for controlling the spread of COVID-19 allowing the rapid identification of active infection and isolation of positive patients. The present revision of the literature has demonstrated that sensitivity and specificity of all available RATs in Spain are high and accomplish European regulations and WHO recommendations. Studies have also shown that these tests are very useful detecting symptomatic patients, preferably less than 7 days of symptoms and ideally less than 5 days. Further studies are required to corroborate the adequacy of RATs for detecting the SARS-CoV-2 infection.

## Funding

This research has not received specific aid from agencies of the public sector, commercial sector or non-profit entities.

## Conflict of interest

None declared.
